# Society as Cause and Cure: The Norms of Transgender Social Medicine

**DOI:** 10.1007/s11013-021-09727-4

**Published:** 2021-06-22

**Authors:** Ketil Slagstad

**Affiliations:** 1 Institute of Health and Society, University of Oslo, Oslo, Norway; 2 Institut für Geschichte der Medizin und Ethik in der Medizin, Charité Universitätsmedizin Berlin, Berlin, Germany

**Keywords:** Transgender history, History of social medicine, History of psychiatry, Medical transition

## Abstract

This article analyzes how trans health was negotiated on the margins of psychiatry from the late 1970s and early 1980s. In this period, a new model of medical transition was established for trans people in Norway. Psychiatrists and other medical doctors as well as psychologists and social workers with a special interest and training in social medicine created a new diagnostic and therapeutic regime in which the social aspects of transitioning took center stage. The article situates this regime in a long Norwegian tradition of social medicine, including the important political role of social medicine in the creation of the postwar welfare state and its scope of addressing and changing the societal structures involved in disease. By using archival material, medical records and oral history interviews with former patients and health professionals, I demonstrate how social aspects not only underpinned diagnostic evaluations but were an integral component of the entire therapeutic regime. Sex reassignment became an integrative way of imagining and practicing psychiatry as social medicine. The article specifically unpacks the social element of these diagnostic and therapeutic approaches in trans medicine. Because the locus of intervention and treatment remained the individual, an approach with subversive potential ended up reproducing the norms that caused illness in the first place: “the social” became a conformist tool to help the patient integrate, adjust to and transform the pathology-producing forces of society.

## Introduction

The history of psychiatry is often told through the metaphor of the pendulum. It is described as a profession that swings between “biological” and “psychological” theories and therapies. Historians of psychiatry, however, have pointed out the limitations and deficiencies of this historiographic lens:^1^ Rather than being two distinct and opposing camps, there are numerous historical examples of interaction, cooperation and mutual learning between advocates of somatic interventions and talking cures (Pickersgill 2010; Rasmussen 2006; Raz 2013; Sadowsky 2005).^2^ By looking beyond this notion of waterproof professional hegemonies and of a pendulum swinging between them, one gains a richer and more complex picture of porous professional “trading zones.”^3^ When the history of psychiatry is reduced to a struggle between psychoanalysts and biologically oriented psychiatrists, the role of “the social” in psychiatry writ large often disappears. Throughout history, mental health professionals have looked to society not only to explain illness, but also to find cures. This article analyzes how self-conscious approaches to psychiatry as a form of social medicine became a mode of clinical practice in the field of trans medicine. Consequently, this article is a contribution to a growing historiography on trans *and* social medicine (Gill-Peterson 2018; Herrn 2005; Holm 2017; Meyer 2015; Meyerowitz 2002; Stryker 2017).

Beginning in the 1950s, following the high-profile and tabloid-scrutinized medical transition of celebrity patient Christine Jorgensen (1926–1989) in Copenhagen, a small number of experts—psychiatrists, endocrinologists and plastic surgeons—in Norway also began to examine and treat people whose gender identities did not match their birth sex. This practice was carried out in an unregulated fashion by experts who exercised fairly free discretion in their therapeutical decisions (Sandal 2017). Because there was no formalized clinic or treatment protocol, the various clinicians approached their patients differently. In Oslo, for example, most trans women were seen by a psychiatrist working in a large hospital who initiated hormone treatment before referring patients to a plastic surgeon for sex reassignment surgery. In contrast, most trans men were seen by an endocrinologist at another hospital who made treatment decisions about hormones and surgical treatment (top surgery) as part of a team of specialists. Trans medicine remained heterogenous and unregulated until the late 1970s. Amid large-scale social movements including the fight for gay and lesbian liberation and second-wave feminism, gay and lesbian medical professionals in Oslo established a counselling service for gays and lesbians as part of the public healthcare service. People requesting medical transition turned to them. Medical professionals interested in sexology and social medicine responded by developing a new diagnostic and therapeutic model for what they called “sex change therapy.”^4^ But during this process, the meaning of social medicine also changed.

This article is based on my dissertation project, which explores the emergence and negotiation of trans medicine in a Scandinavian context in the second half of the twentieth century. The research project examines a range of material, including unpublished sources from public archives (e.g., ministries and directorates), private archives (e.g., of trans people, activists and health professionals), medical records (patients who were assessed from the 1960s to the early 2000s) and more than twenty oral history interviews with former patients, activists and health professionals. In this article, I use selected findings to show how tools from social medicine were integrated into the diagnostic and therapeutic regime of medical transition, what I define as “trans social medicine.” The paper complicates a historiography of social medicine and the Norwegian public health system that has tended to focus on grand politics and political ideology and that has often applied a top-down perspective to the organizational structure of the health bureaucracy (see, e.g., Berg 2009; Schiøtz 2003b; Slagstad 1998; but see Schiøtz 2003a). The article concludes with a consideration of this specific historical example’s implications about the broader relationship between psychiatry and social medicine.

## Psychiatry as Social Medicine

Social medicine is a term with different meanings in different places (Pentecost et al. 2021), but in Norway, as in most of Scandinavia, social medicine was a key element in the creation of the postwar welfare state. One of the staunchest advocates and implementers of social medicine in Norway was Karl Evang (1902–1981), the powerful director general of health for more than three decades, from 1938–1972.^5^ He believed the state should implement medicine for the public good and defended a health policy rooted in science and social science. Sexuality played a major role in his comprehensive concept of health: He argued that societal structures, especially religion and capitalism, prevented “natural sexuality” from developing freely. He believed that free sexuality, in turn, would be good for society (Berg 2002:115–117; Nordby 1989:59–65; Jordåen 2020). Society’s role in health and disease was at the heart of the health administration’s visions of public health, especially when it came to prevention. According to the principles of social medicine, at least as conceived by the Directorate of Health, medicine and politics were inseparable.

Evang’s views resonated outside Norway. The World Health Organization (WHO) had defined health in its 1946 constitution as “a state of complete physical, mental and social well-being and not merely the absence of disease or infirmity.” As one of the architects of the WHO, Evang remained faithful to a comprehensive concept of health [*et utvidet helsebegrep*] in which people and their environments were seen as inseparable. Health and disease depended on cultural, societal as well as biological factors, and thus, knowledge about the body, psyche and humans *in* society were brought together to form a holistic understanding of health (Berg 2002:15). While Evang, in a 1971 article, praised the concept of “psychosomatic illness” as a step in the right direction, because it broke with a dualistic “Western” understanding of the separation between the body and psyche, he criticized the concept for overlooking the social component of health and illness (Evang 1971:40). He had already emphasized the pathogenic role of social and economic structures in the early 1930s (Evang 1931): In social medicine, the social was an integral part of disease ontology.

In Norway, social medicine was anchored in the academy, in medical education and clinical practice. When the University of Oslo’s Institute of Hygiene was divided into one institute for hygiene and one for social medicine in 1951, the country’s first professorship in social medicine was established.^6^ In his textbook for medical students, Professor Axel Strøm (1901–1985) defined social medicine as the subject concerned with “the reciprocal relationship between society and health” (Strøm 1956:12).^7^ Strøm sought to broaden the horizon of social medicine beyond its primary concern with health at the population level to include perspectives and tools for clinical practice as well. Social medicine became part of the medical curriculum and a handbook of social medicine for clinicians was published in 1955 (Marthinsen et al. 1955), and, three years later, social medicine became a proper clinical specialty.

Social medicine itself underwent a radical transformation from the interwar and postwar periods to the 1970s. It shifted from being closely tied to hygiene and the problem of infectious diseases in the interwar period to being increasingly preoccupied with social benefits and care for minorities, people with disabilities and social “outcasts.” The professionals involved in social medicine saw this as a shift in perspective from “the macro to the micro”—from the population level to that of vulnerable groups (Strøm et al. 1973). This change coincided with the introduction of a new kind of expertise into the sphere of social medicine: psychiatry. Social medicine, which had previously been dominated by specialists in infectious disease and public hygiene, was increasingly taken over by psychiatrists. From the 1970s onwards, all chair positions in social medicine in Oslo were filled by psychiatrists.

Although experts in social medicine stressed the importance of making decisions on an objective basis precisely because their profession was so closely linked to politics, it would be misleading to see this as a call for the separation of science from politics.^8^ The experts instead believed “objectivity” had to be pursued *because* society, politics and medicine were inextricably linked:

From its beginning, social medicine has been influenced by political ideology in society and it has itself contributed to shaping political ideas. There is thus a close relationship between social medicine and social policy. … It is impossible for a professional of social medicine who wants to change the inequalities he [*sic*] has pointed out not to be politically involved. … In political action, however, he must be free to choose (Strøm et al. 1973:14).^9^

While social medicine gained importance in public health thinking, two trends ran counter to this development: the specialization and the democratization of medicine. In the postwar decades and especially in the 1960s and 1970s, the number of doctors, and especially hospital specialists, increased per capita. With larger hospitals and increasingly advanced hospital medicine, more and more doctors worked in hospitals (Haave 2011:83–96). As medicine became more specialized, the profession fragmented into smaller and smaller subfields. This threatened a holistic understanding of health and disease, including of the interactions between society and patient—from cause to cure.

The specialization and professionalization of medicine may have also caused the medico-political framework of social medicine to fall out of favor. The director general of health himself admitted that the comprehensive concept of health had not caught on, either with the public or with politicians and that even medical colleagues clung to “the old primitive concept of health” (Evang 1971:40–41). The editor of a 1981 textbook on social medicine deplored the “aversion to knowledge of the environment and society’s influence on individual dysfunction” (Sundby 1981:19). He criticized the medical profession for being “dominated by a predominantly curative and reparative medical tradition” but more importantly argued that the ideas of social medicine had not gained a strong foothold in society (ibid.).^10^ The professor of social medicine had come to the sad realization that “social medicine so far had little influence on the client producing society” (ibid.).^11^

During the same period, from the 1970s onwards, the public system of health councils run by state-employed physicians came under pressure from politicians and activists who wanted a more democratic and less hierarchical system (Schiøtz Schiøtz 2003a:351–360). In 1983, a new healthcare act terminated the role of the government-appointed district medical officer. Three years later, the social medicine specialty was dissolved. Finally, by the late 1980s, the health councils were dismantled.

## The Oslo and Baltimore Model of Gender Identity Formation

In the late 1970s and early 1980s, the Oslo Health Council became a laboratory for experimentation with sexology and the practical application of social medicine theory.^12^ The head of the council, Fredrik Mellbye (1917–1999), had served as the chief medical officer at the Directorate of Health’s Office of Hygiene and was a strong believer in social medicine’s potential for improving the health of the population. In 1977, a group of gay and lesbian medical professionals established a counselling service for homosexuals in the Oslo Health Council. In clinical practice and among colleagues, they were confronted by the lack of knowledge about and training in gay and lesbian health. More generally, they had witnessed the negative health effects of homophobia in the healthcare system and society. Numerous patients did not dare to talk openly about sex and sexuality with general practitioners. Many had experienced discrimination and harassment by healthcare workers, and 75 percent of patients presented some sort of social or sexual issue or problems related to their acceptance of their own sexuality (Slagstad 2020). Soon, people requesting medical transition also sought their help.^13^

The gay and lesbian medical professionals and counsellors were supervised by psychiatrists and psychologists. Among them was Berthold Grünfeld (1932–2007). Under his leadership, Norway’s first department of medical sexology was established at the Oslo Health Council in 1979.^14^ After studying medicine and becoming a specialist in psychiatry, he devoted the rest of his life to forensic psychiatry and social medicine, in which he later became a professor. By combining sexology with social medicine, Grünfeld’s approach to health problems became of paramount importance to the therapeutic regime of medical transition from the late 1970s onwards.

For Grünfeld, sexuality was a fundamental dimension of human life, and therefore “good” sexual health was an integral part of a comprehensive concept of health: “[Sexual health] is a resource not only to be preserved but also to be used” (Grünfeld 1979:168).^15^ Sexuality, he argued, “permeates us and shapes our motives and actions far more comprehensively than what we are accustomed to associating with procreation” (ibid., 11).^16^ Sexuality is “a primal force” [*urkraft*], Grünfeld thought, “the more one tries to suppress it, the greater concern it becomes. Suppression dehumanizes it, turns it into something dirty and sleazy, something we are ashamed of. Unfortunately, our culture has far too much of this destructive attitude towards sexuality” (ibid., 114).^17^

**Fig. 1:**
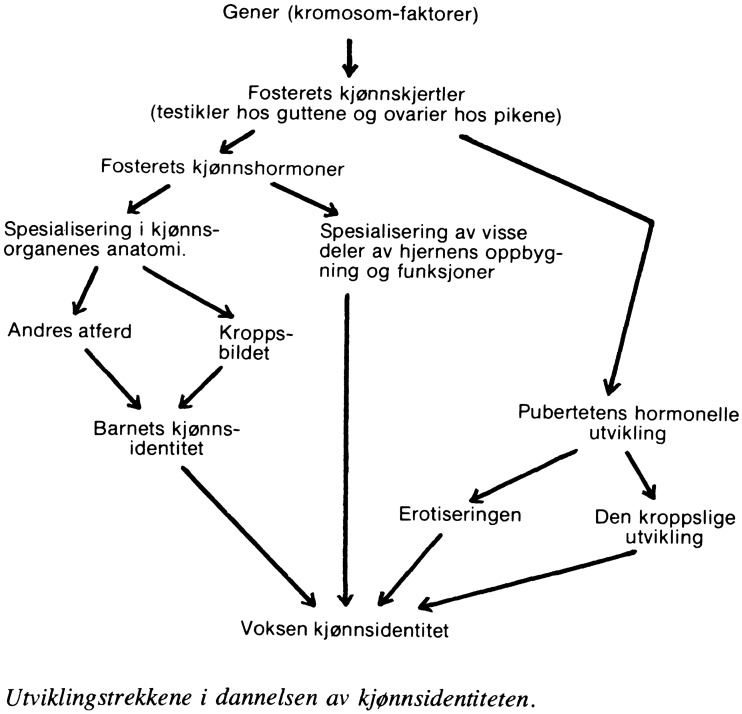
Flow chart of gender identity development. In Berthold Grünfeld, *Vårt seksuelle liv*, 1979. Courtesy of Gyldendal forlag.

Grünfeld’s thinking resonated with what Evang wrote earlier: “There is nowhere for the coming generation to get sound and true information about human sexual life,” the socialist doctors deplored (Evang et al. 1932).^18^ For Grünfeld, sexuality and gender identity were products of biological, psychological and social stimuli, and many developmental steps were part of an ontogenetic model of gender identity formation: from genes and fetal hormones through puberty, body image and environmental factors (Fig. 1).

This model had strong similarities to the Baltimore model of sex-gender development constructed by psychologists working with intersex children at Johns Hopkins University in the 1950s and 1960s. These included John Money (1921–2006), who would become a seminal figure in the history of intersex conditions and transsexualism (Eder 2010; Downing et al. 2014) and who developed a complex theory of the plasticity of gender identity formation in children (Gill-Peterson 2018:97–127). Indeed, Grünfeld’s chart of gender identity development was a blueprint of the Baltimore model.

According to Grünfeld, only two sexes and genders^19^ were conceivable—and possible: “Gender roles, gender identity, hormones and other factors go together and gradually create man and woman, the adult psycho-sexual personality” (Grünfeld 1979, 26).^20^ In a similar vein, the Baltimore psychologists argued: “Alone among the diverse functional systems of embryonic development, the reproductive system is sexually dimorphic. Thus, also in subsequent behavioral and psychic development, there is sexual dimorphism” (Money and Ehrhardt 1972:1). The axiom of the Baltimore model was that the “successful” formation of gender identity in children depended on the process of “complementation to members of the opposite sex, and identification with members of the same sex” (ibid., 13). The formation of gender identity was seen as equivalent to the infant’s process of learning a language, i.e., there existed a biological program upon which gender differences had to be learned. However, this plasticity was limited to the first eighteen months after birth, after which the window of imprinting closed.

Of paramount importance to this learning process was human “dimorphism”: for the child to develop a “secure” gender identity as a man or woman, the mother and father had to be *distinguishable* from each other, as manifested in anatomy (external genitals), physique, depth of voice, etc. Following this reasoning, trans people who had gone through hormonal and surgical transition could become parents as long as their “outward appearance” was easily distinguishable as man or woman and they fulfilled the traditional role as husband and wife (ibid., 14). As Jennifer Germon has pointed out, Money situated the intersexed body as “unfinished and finish-able,” with medical technologies promising to restore what nature had not completed: “When perceived as unfinished—or disordered—in a context where interventions are possible, medical science is compelled to make an intervention, to make things right, to ‘finish what nature failed to do,’ to bring order” (Germon 2009:55). In other words, the entire theory of gender identity formation, in both the Baltimore and Oslo models, not only relied on but also supported and maintained a dichotomous understanding of sex and gender.

The model of gender developed for intersex was extended into a biosocial framework for gender development in general: the multifactorial ontogenetic model of gender identity enabled a possibility of gender identity not always developing congruently with sex assigned at birth. In other words, gender identity, defined by Grünfeld as “psychological sex,” could divert from “biological sex”: “Usually, there is congruence between the biological and the psychological sexes [*kjønn*]. But sometimes there is conflict between the two” (Grünfeld 1979:21).^21^ This model went hand in hand with the developmental model of Professor Richard Stoller (1924–1991), a psychoanalyst and psychiatrist at the UCLA Gender Identity Clinic, who in the 1960s famously coined the concept of “core gender identity.” He argued the child’s core gender identity was learned postnatally and primarily “culturally determined” by “the infant-parents relationship, by the child’s perception of its external genitalia, and by a biologic force that springs from the biologic variables of sex. … The first two factors are almost always crucial in determining the ultimate gender identity” (Stoller 1968:30). Underpinning his model was the idea of symbiosis with and separation from the mother: While girls did not have to “surmount” their relationship with their mother, the “successful” development of a male identity in boys—“becoming a separate masculine individual”—hinged on breaking away from “profound identifications with his mother” (Stoller 1968:263–264). Although gender identity was not fully developed until the end of adolescence, Stoller concluded that the formation of the core gender identity was complete before the phallic state. The notion of the immutability of gender identity became an axiom and fundamental justification for medical transition in the first place: the medical model of variation in sex-gender as “development gone awry” and the notion of gender identity as fixed—after childhood—were fundamental to the treatment regime that grew out of the Oslo Health Council in the late 1970s and early 1980s, similar to the reasoning of Money and Stoller.

However, Money, Stoller and Grünfeld’s approaches differed significantly in where they situated “the social.” The social in the Baltimore model was limited to the socialization—or “imprinting”—of the child within the family; a model of psychosexual differentiation heavily influenced by behaviorist thinking (Repo 2016:24–48). For Stoller, a psychoanalyst, the social was limited to the dynamics within the nuclear family (Germon 2009:65–66) and “funneled through the mother” (Stoller 1968:xi). For Grünfeld, on the other hand, the role of society in the formation of gender roles and identities was of “colossal importance,” whether it was at school, among friends, in sports, at home, in mass media, books, film, TV—“everywhere”: “the signals are constantly there giving messages. … The role pattern is clear and unambiguous. It tells you what you may and may not do. Breaking the laws is punished” (Grünfeld 1979:24).^22^ Grünfeld mocked society’s restraining and conservative norms of sexuality and gender, which required boys to be tough and never express feelings of sadness in public and girls to be polite, cute and neat. School textbooks reproduced stereotypical ideas of mothers as weak but kind housewives and the strong and strict father as the family breadwinner: “The message is that women are more helpless, weaker and inferior to men. This diffuses into the child mind like hot cake with jam sliding down the throat of a hungry kid. And it stays there in the mind, it is stored” (ibid., 25).^23^ Society was at the heart of the Oslo Health Council model of gender identity formation, its norms infused into people’s minds.

## The Social of Diagnostics

In the late 1970s, medical professionals in the Oslo Health Council laid the groundwork for the future diagnostic regime and treatment protocol for medical transition for trans people. While some colleagues—psychiatrists and psychologists—argued that patients could be helped through psychotherapy, the professionals who worked at the council were convinced that after puberty, gender identity could not be changed through, for example, psychotherapy. “We assume that a person’s gender identity is decisively fixed when the child starts in school. At this point, the identity is so fixed that it cannot be changed” (Grünfeld 1979:21).^24^ Therefore, gender dysphoria had to be resolved by other means, usually hormones and surgery.^25^ The head of the sexology department wrote that it was his duty as a doctor to help people live better lives, based on their “subjective experience of their situation.”^26^

But the professionals thought that not everyone who applied for medical transition would benefit; the criteria had to be strict, and treatment would be granted only to “well-selected cases.” Trans people, who until then had faced various diagnostic and therapeutic regimes depending on which professionals they saw, were subjected to thorough inspection and adjustment grounded in sexology and social medicine in keeping with the Oslo model. The diagnostic process lasted six to twelve months. No hormonal therapy was initiated during this time. All diagnostic and therapeutic decisions were anchored in a team of experts (psychiatrists, experts of social medicine, sexologists, endocrinologists, social workers, psychologists and plastic surgeons), all professionals were involved early in the diagnostic process with the client, and therapeutic decision were based on “the views of all the aforementioned professions.”^27^ Making decisions in a team allowed each case to be scrutinized from a range of biological, psychological and social perspectives. For example, a physician or surgeon carried out a thorough clinical examination including all aspects of the clients’ “somatic sex” to exclude genetic, hormonal or genital “incongruences.”

Two criteria were fundamental to treatment: a person had to be diagnosed as a “true transsexual” and there could be no contraindications. The diagnostic criteria for transsexualism were the same as in neighboring Sweden, and the criteria mirrored those of the ICD-9, published in 1978: since childhood, patients had to have had the experience of “belonging to the opposite sex” and “feelings of disgust” with their own “own biological sex” as well as a desire to be recognized as the “opposite sex” and a desire for hormonal and surgical therapy to align the body with their gender identity.^28^

Internalized homophobia, relational problems, sexual fantasies, coming-out processes and minority stigma were commonplace issues for gay and lesbian health professionals at the Oslo Health Council. Confronted with the new clinical problem of medical transition, they brought this knowledge with them, raising questions: How did narrow societal norms of gender affect lives and self-understandings; who decided what counted as masculinity and femininity, and to what extent were these characteristics dependent on social norms; what significance did sex play in their clients’ lives; and how did patients imagine sex after medical transition?

The likelihood of successful “adjustment” after “conversion” increased if the social stability was good before treatment, the Oslo Health Council stated.^29^ These factors were not treated as individual and separable, but as interwoven and co-constitutive. A good surgical result was of little use if the social situation was unstable and the patient had to be sufficiently psychologically robust to handle the hormonal and surgical therapy. The numerous contraindications to treatment also highlighted the importance of social aspects in the decision-making process: lack of resources, poor social situation [*ressurssvak sosial situasjon*] or lack of support from friends and family were important contraindications, as were complicating sexological aspects, “advanced age” and “unsuitable physique” [*uegnet kroppsbygning*].^30^ At each level, the patient’s identity and desire to transition was seen in relation to society.

At first glance, it might be tempting to interpret the criteria of a “suitable” physique or body characteristics as evidence of an underlying stereotypical and normative notion of what constituted a man or a woman. One of the physicians who worked in the council confirmed this notion:

One of the criteria for sex change, which was very strict, was that one had to be able to pass as the other sex [*kjønn*]. That’s why tall men didn’t get treatment and people with big shoes. … I remember very well how this passing criterion was talked about. Talk about cultural production of masculinity and femininity and what is right and wrong and normal and abnormal. It’s very strange to think about today, I think.^31^

Hanna, who transitioned in the 1980s, recalled in an interview that her impression was that “it was a very conservative opinion of how a woman should look and be. I know of several people who were stopped because their appearance was a little too masculine. Those who were too tall were stopped.”^32^ The physical criteria reflected the thinking of the endocrinologist and seminal figure in the history of transsexualism in the United States, Harry Benjamin (1885–1986). Benjamin’s approval of sex reassignment surgery for trans women largely depended on whether he envisioned the person as a “successful woman.” He argued that “the outward appearance and the impression of the total personality” were crucial: “A heavy masculine build, a height of six feet or more, and a strong, dark beard were causes for worry and doubt,” Benjamin wrote in his seminal book, *The Transsexual Phenomenon*. Still, “even with these handicaps,” he added, “the operation was performed in several instances, with or without my consent. So far, all seems to have gone well with them” (Benjamin 1966:137). Stoller also concluded that given the lack of proper follow-up studies and prognostic criteria only “those males [*sic*] who are the most feminine” should be offered medical transition (Stoller 1973:251).

The medical records of the Oslo Health Council, however, show that stature and physique were not per se determinants of who got access to treatment. For example, one patient whom the surgeon described as “somatically not immediately convincing with a sturdy physique including a large, coarse face with much dark colored facial hair,” received hormones and surgery because she had been “observed and assessed over several years”: “it seems obvious that she has definitely chosen the female role and can fulfil this and therefore it seems right to help with surgery.”^33^ Another patient who was also assigned male sex at birth, who was over 1.90 meter tall and had a shoe size of 48, also had surgery, a psychologist involved in the program told me.^34^

These criteria not only reflected narrow gender roles and ideals in the thinking of medical professionals but expressed the normative basis of social medicine in the therapeutic regime. Although the treatment protocol undoubtedly reproduced gender binaries and foreclosed diverse gender expression, the therapeutic goal per se was not to reproduce stereotypical, gendered phenotypes. On the contrary, the professionals envisioned treatment goals *in relation to society*: After treatment, the patient had to adapt to, adjust to and integrate into society. Because a comprehensive concept of health relied so heavily on seeing health and disease as an interaction between the products of biological, psychological and societal factors, this also meant that the clinicians had to envision how the person would integrate into a society with narrow norms of gender expression. The goal of the diagnostic regime seemed to be less about identifying who could fulfill narrow bodily norms of masculinity or femininity and more about producing a “social medical norm of gender” that included traditional societal ideas about gender roles. Importantly, the site of intervention remained the individual, who had to absorb, adjust to and transform societal norms. The Oslo model of trans social medicine was an apparatus for “improved adjustment” to society, as the guideline put it, rather than a critique of the norms which produced pathology in the first place.^35^ Thus, paradoxically, a potentially subversive technique was predicated on reinforcing established narrow gender norms.

## Integrating Diagnostics and Therapeutics

Once a person was deemed ready to begin medical transition, preoperative “conversion” took place on several levels concomitantly: hormonally, mentally, socially. A crucial goal was to firmly integrate the clients into their “new” gender expression *in society.* Professionals helped facilitate social transition in the workplace, even by arranging for occupational rehabilitation [*attføring*] or the relocation to a new job. This policy was not only in line with postwar welfare policies aimed at securing the population’s labor force, but also reflected the important role of labor participation in social medicine’s comprehensive concept of health.

The following example is significant. “The client is in a very complicated situation psychologically and socially. He [*sic*] has from childhood had the need to dress and appear like a woman. For this reason, he has found a foothold in a work situation or situation that has given him general social status/acceptance,” the professionals wrote about a trans woman who sought their help in the late 1970s.^36^ Because of the complexity of the situation, the doctor and social worker concluded, a series of interventions needed to be implemented that should begin with “clarifying what his options are for vocational rehabilitation/occupation.”^37^ In other words, integration into work life was not seen as separate from or as a prerequisite for access to medical transition, but as an integral part of the therapeutic process.^38^

The new phase of preoperative transition lasted another 6 to 12 months and required the patient to be closely supported and monitored while major physical and life changes took place. From this point on, the professionals demanded that the patient live full-time in the expression of “the opposite gender [*kjønn*].” The therapeutic regime of social medicine required that patients come out to families and colleagues early in the process of medical transition. Hanna recalled in an interview: “One of the things the doctors stressed, they only cared about one thing, and that was whether your family supported you. Even the surgeon asked what my mum, dad and siblings said. … Grünfeld wouldn’t even have considered me as a patient if I hadn’t come out. There was no way around it.”^39^ Doctors sought not only to test the person’s conviction or determination; they also aimed to anchor the transition process in the person’s environment and social life. Hanna said,

I got the impression that he did not want to be blamed if my family turned away from me when I hadn’t made an attempt to do something. The social had an enormous value to him, so in our conversations, he kept coming back to what I had done with my family, vacations with my siblings visiting. … He emphasized your social life as a woman to an extreme degree. That’s where he probed how I handled my life, my daily routines and my free time.^40^

The professionals didn’t just want to safeguard decisions to reduce the risk of future regret, but this example also shows how social life was integrated into the therapeutic process.

As diagnostic assessments and therapeutic decisions became interwoven, psychological and social aspects of transitioning also became inseparable. This was evident, not least, in the experts’ reflections on the name change during the transition process. One psychologist, who was asked to provide expert advice when the team was in doubt about treatment decisions, recalled how difficult the position could be: “I found it very hard to say no. Who was I to make decisions in their lives?”^41^ The psychologist recalled having many conversations with clients about choosing a name. “It was important to me that they should not become too outré, too over-the-top. Often, they wanted names like Sonja and Sylvia, but I tried to convince them to choose names closer to the ones they already had.”^42^

On the one hand, the desire to adopt traditional gendered (and royal) names was an expression of narrow gender ideals and norms in society, and these names probably signaled a flamboyant kind of celebrity femininity that was far removed from the social class and everyday life in which the patients lived their lives.^43^ But for the psychologist, it was also important from a professional perspective to facilitate the integration of past life history into the therapeutic approach and treatment goals: “In a way, I wanted them to keep some of their identity. It was a little sad if they thought that it was going to be a complete transformation. I thought they were carrying a lot of value with them, that they weren’t going to be completely new.”^44^ In trans social medicine, diagnostics became inseparable from therapeutic reasoning. “He wanted us to bring with us the whole human being. We were supposed to carry our whole story with us. Everything that you brought with you, you weren’t supposed to leave anything behind,” Hanna said with reference to Grünfeld.^45^ Social medical norms of gender, including the importance of integrating past experiences with a future gendered self *in* society were often irreconcilable with patient’s own desires, experiences and coming out processes.

## Social Medicine as an Ambivalent Form of Care

This article has unpacked “the social” in the diagnostic and therapeutic regime of medical transition in Norway from the late 1970s and early 1980s, a time when new diagnostic and therapeutic practices were developed based on social medicine. Social medicine became the main theoretical basis for this, not only as a model for how gender identity developed in interaction with society, but also as a model for the intertwined diagnostic-therapeutic approach to medical transition. These approaches partly evoked a longer tradition of promoting social medicine in the Norwegian health bureaucracy, including a comprehensive concept of health. But they also emerged through the Oslo Health Council’s experimentation with the tools of social medicine. The Oslo Health Council was dissolved in 1988 as part of a reorganization of the public health system. Grünfeld continued to work with trans patients until the early 2000s, but when he retired, trans social medicine vanished. In 2002, a national gender identity clinic was established at the National Hospital in Oslo under the direction of psychiatrists, a clinic that to this day exercises a monopoly over gender affirming therapy in Norway.^46^

Trans social medicine was not so much a revival as it was a reshaping of an old field of knowledge confronted with a new clinical problem. Although social medicine had a long tradition of caring for marginalized groups in society and promoting the importance of sexuality in a comprehensive concept of health, social medicine was also anchored in paternalistic and heteronormative medical traditions. This was evident not least in public responses to AIDS, a major public health crisis that also unfolded in the 1980s. Public responses to AIDS were grounded in social medicine, but activists challenged paternalistic and homophobic tendencies in public health thinking, reshaping the face of social medicine (Slagstad 2020). In the 1980s, however, there were no activist groups advocating for trans care that could mobilize media and authorities as AIDS activists successfully had.^47^ Grünfeld was himself aware of the normative and paternalistic approach to trans people in social medicine: the paternalism of medical professionals making decisions for patients seeking medical transition often remained unconscious, he wrote, “disguised as so-called medical reasoning” (Grünfeld 1987:203).^48^

However, the therapeutic regime of social medicine established in the late 1970s and 1980s was also an attempt to safeguard medical decision-making in a field where scientific knowledge was sparse and experts lacked clinical experience. The professionals themselves justified the long observation time and restrictive approach to therapy as a kind of care—primarily caring to do good by doing no harm. As one psychologist said in an interview, “I felt very strongly that I or we cared about the patients’ situation, their feelings, their integrity, that you shouldn’t make bad matters worse, that you shouldn’t start anything without a proper foundation.”^49^

Care implies more than the promotion of patient autonomy and choice, argued the philosopher of medicine Annemarie Mol, claiming that the antipode of the logic of care is neglect (Mol 2008:xi). Ingrained in the logic of care is an ethical framework grounded in practice, a different ethics from the argumentative and analytical approaches of bioethics.^50^ In the “discursive regime” of bioethics that grew out of the 1960s and 1970s, which enacted informed consent and patient autonomy as the core values of good practice, the “social subject” was replaced by “the self,” wrote the historian of medicine Roger Cooter (Cooter 2010).^51^ Measured against Mol and Cooter’s analytical framework, the Oslo model of medical transition was a strict, restrictive, paternalistic and normative, but also pragmatic, cautious and engaged form of care that sought to mediate between the social subject and the self.

The professionals at the Oslo Health Council interpreted their patients’ experiences of dysphoria or desire to transition as a product of narrow social norms: “Our clients describe a strong despair about living in a society where women are not allowed to look like men and vice versa. For our clients, these limits are so narrow that only an operation can solve the conflict between the expectation of others and their own behavior” (Malterud and Solberg 1983).^52^ They argued that medicine should not become a “quick fix” to structural and societal problems, reflecting the long tradition in social medicine of skepticism about the use of pharmaceuticals and “medicalization” of life problems (cf. Hobæk and Lie 2019). Professionals had seen how narrow norms of sexuality and gender produced illness in their homosexual patients and in their own lives as gays and lesbians. Partly inspired by Thomas Szasz and Janice G. Raymond’s critique of medicalization, which some of the professionals referred to in their article published in the *Journal of the Norwegian Medical Association*, “sex change therapy” was seen as “supporting the narrow limits of gender role behavior and the strict requirements of gender role conformity” (Malterud and Solberg 1983).^53^ Unlike homosexuality, trans (or indeed transsexualism) was not seen as a minority condition oppressed by the same narrow societal norms as other minorities. Following the logic of social medicine, restricting access to medical interventions for trans people became a way to combat patriarchal structures and disrupt restrictive norms of gender and sexuality. Although professionals recognized how the stigma against and marginalization of trans people led to mental health issues such as depression, their intervention focused on the individual rather than society. The goal of the Oslo medical transition apparatus was to reintegrate the patient into society.

As a result, the diagnostic and therapeutic apparatus of social medicine paradoxically reproduced the same narrow gender norms it sought to dissolve: its ontogenetic model of sex and gender identity formation was based on dimorphism, as the diagnostic criteria for “true transsexualism” reproduced binary and stereotypical conceptions of gender and no treatment, such as hormones, was offered unless the patient underwent a full surgical transition. As Dallas Denny wrote of U.S. gender identity clinics, the criteria of passing “forced unrealistic stereotypes of femininity and masculinity on transsexual men and women” (Denny 1992:12). The medical model of transsexualism tended to reproduce its own epistemological foundation. “Who is telling the story for whom, and how do the storytellers differentiate between the story they tell and the story they hear?” wrote Sandy Stone in her pivotal essay, “The *Empire* Strikes Back: A Posttranssexual Manifesto,” in which she rebuked the anti-trans position of Janice G. Raymond (Stone 1991). The medical apparatus of transsexualism had become a monolithic model of subjectivity formation, the clinic “a technology of inscription” and its clinicians “gatekeepers for cultural norms,” ultimately preventing the emergence of a plurality of trans embodiments and subjectivities (ibid.).

As much as this story shows how this practice was rooted in and produced new social medical norms of gender, it is also a recent historical example of integrative thinking and of the practicing of psychiatry as social medicine. This chapter of history demonstrates the increasing influence of social medicine on psychiatry—and vice versa. As psychiatrists took over the chairs in social medicine, the field, which had deep ties to hygiene, was remade into a new version of social medicine centered around marginalized and stigmatized groups, including people with drug and alcohol addictions, poor people, sex workers, the unemployed, sexual minorities—and trans patients. This new version of social medicine differed from the postwar Latin American versions of social medicine, which were dominated by a Marxist approach and revolutionary goals. In many Latin American countries, Marxist and post-Marxist social and political theory was fused with medicine to pursue the radical goal of transforming society (Porter 2006). However, the Latin American and Norwegian version of social medicine had commonalities in the sense that the profession was anchored in the academy and in medical education.

But the Norwegian version of social medicine also differed from the Anglo-American context and from the latter’s approach to public health. In the Anglo-American version of public health, populations were visualized as a “sum of individuals” (Waitzkin et al. 2001). In the United States and the United Kingdom, this led to an increased focus on risk factors and on individuals’ responsibilities for their own health, propelled by the rise of epidemiology and “risk factor thinking.” This was evident not least in the medical construction of smoking as an individual’s risk behavior and responsibility, an epistemology that ignored cultural and socioeconomic factors.^54^ Dorothy Porter has argued that in postwar Britain, the rise of socio-behavioral studies initiated a shift in thinking in social medicine from *life conditions* to *lifestyles,* an analytical shift from social structure to social behavior and towards individual unhealthy behavior being constructed as the primary cause of noncommunicable diseases (Porter 2006, 2011:159–174). Smoking and lung cancer became successful epidemiological models for a biopsychosocial approach to health and disease, while simultaneously grounding the model in an individualistic basis of social medicine.

This version of social medicine focused on prevention of disease through lifestyle and behavioral changes, but ultimately underscored “the individualist focus of therapeutic medicine” (Porter 2006; see also Porter 2011:205–207). Medicine became more biomedical, coinciding with the rise of epidemiology and advanced multivariate statistical analyses, and in the postwar United States, public health became “increasingly accommodationist to the authority of biomedicine,” argued Allan M. Brandt and Martha Gardner (Brandt and Gardner 2000). Whereas the Anglo-American version of public health turned to the individual to explain differences in health,^55^ the Latin American and Norwegian versions of social medicine approached public health by viewing populations as social institutions within a political framework. In the latter versions of social medicine, categories like social class, culture, economic production, participation in the labor market and gender differences served as prisms to analyze health disparities.

Perhaps the Norwegian version of social medicine of the 1970s and 1980s can best be described as a third way, an approach that mediated between the Latin American and the Anglo-American version of social medicine, constantly moving between the population and the individual. The case of medical transition illustrates how psychiatrists and specialists in social medicine attempted to create a form of medicine that cared for the patient *in* society. In this form of medicine, society and societal norms were at the heart of disease, its cause and its cure. Paradoxically, a profession dedicated to caring for society’s outcasts became the primary gatekeeping institution for trans healthcare: The social element of social medicine became the greatest barrier for trans people trying to access hormonal and surgical therapy.

“What if we’re not trapped in the wrong body but trapped in the wrong society?” wrote Juliet Jacques (Jacques 2015:341). But what trans people viewed as the wrong society was not the same as what social medicine regarded as the wrong society. Instead of confronting the pathology-producing norms of society, trans people had to conform to the norms of social medicine.

## Footnotes

1 For accounts of “biological psychiatry,” see for instance (Braslow 1997; Healy 2002; Shorter 1997).

2 Psychoanalysis as a therapeutic method did not gain a strong foothold in Norwegian psychiatry until the postwar decades, but nevertheless served as an important theoretical resource for psychiatrists earlier in the century. See (Haave 2003, 2008).

3 For the concept of the “trading zone,” see (Galison 1997).

4 The Norwegian terms in use were *kjønnsskiftebehandling* and *kjønnskorrigerende behandling*.

5 Before 1945, the title was “medical director” [*medisinaldirektør*].

6 The new profession of social medicine, however, built on the broad field of hygiene. See (Larsen 2016).

7 “Sosialmedisinen beskjeftiger seg med det gjensidige forhold mellom samfunn og helse.”

8 For the argument that experts called for this separation, see (Larsen 2016).

9 “Fra sin første begynnelse av har sosialmedisinen blitt preget av den politiske ideologi i samfunnet, og den har selv bidratt til å utforme de politiske idéer. En nær relasjon mellom sosialmedisinen og sosialpolitikken er derfor til stede … En sosialmedisiner som ønsker å få rettet på misforhold som han har påvist, kan derfor ikke unngå å bli engasjert i sosialpolitikk. … Men i valg av politiske løsninger må han stå fritt.”

10 “… at det er en uvilje til stede mot kunnskap om miljøets og samfunnets medvirkning til individers funksjonssvikt … dels fordi vi preges av en behandlingstradisjon som overveiende er kurativ og reparativ, men hovedsakelig fordi verken sosialmedisinske eller medisinske synspunkter og interesser i seg selv har særlig vekt i det spill av krefter som former samfunnet.”

11 “… hittil har sosialmedisinen hatt liten innvirkning tilbake på det klientskapende samfunnet.”

12 The role of sexology will be fleshed out in another manuscript by the author.

13 Calle Almedal, Kirsti Malterud, Åse Brit Johnsen and Georg Petersen, “Foreløpig rapport fra Rådgivningstjenesten for homofile” [Preliminary report by the Counselling Service for Homosexuals], 18 May 1978. Oslo helseråd [The Oslo Health Council], box 122, Homofile–transseksualitet, Oslo City Archives, Oslo (hereafter OCA); “Årsrapport for Oslo helseråds rådgivningstjeneste for homofile, erfaringer og vurdering etter ett års prøveprosjekt, november 1977 til november 1978” [Annual report by the Counselling Service for Homosexuals], 15–18, Skeivt Arkiv, Bergen.

14 Hans Døvik, chief medical officer in Oslo, “Rådgivningstjenesten for homofile – egen seksjon for medisinsk sexologi” [New section for medical sexology], 3 July 1979, Oslo helseråd, box 122, Homofile – transseksualitet, OCA.

15 “[Seksuell helse] er en ressurs man ikke bare skal ta vare på, men bruke.”

16 “… gjennomsyrer oss og preger våre motiver og handlinger langt utover det vi vanligvis er tilbøyelige til å forbinde med forplantningen.”

17 “Jo mer man prøver å undertrykke den, desto mer blir den til en plage. Gjennom undertrykkelsen avhumaniseres den, gjøres til noe skittent og tarvelig, noe vi skammer oss over. Og dessverre inneholder vår kultur fortsatt altfor mye av denne destruktive holdningen til seksualiteten.”

18 “Den oppvoksende slekt får ingen steds sunn og sannferdig opplysning om menneskets seksualliv.”

19 In the Norwegian language, there is only one term for sex and gender—*kjønn.*

20 “Kjønnsroller, kjønnsidentitet, hormoner og andre faktorer går sammen til en helhet og skaper gradvis mannen og kvinnen, den voksne psyko-seksuelle personligheten.”

21 “Stort sett er det samsvar mellom biologisk og psykologisk kjønn. Men i enkelte tilfeller oppstår det konflikter mellom de to.”

22 “Signalene er hele tiden til stede og gir beskjeder. … Rollemønsteret er klart og entydig. Det forteller hva man har lov til og hva man ikke har lov til. Å bryte lover straffer seg.”

23 “Og budskapets innhold er at kvinner er mer hjelpeløse enn menn, svakere og underlegne i forhold til mennene. Det går inn i barnesinnet som varmt hvetebrød med syltetøy glir ned i halsen på en sulten unge. Og det blir der i sinnet, det lagres.”

24 “Vi regner med at individets kjønnsidentitet er endelig fastlagt når barnet begynner på skolen. Da er identiteten så fastgrodd i personligheten at det ikke er mulig å endre den.”

25 The Oslo Health Council, December 1979, “Utredning om transseksualitet” [Report on transsexuality], 2, Oslo helseråd, box 122, Homofile – transseksualitet, OCA. “For genuint transseksuelle individer har man erfart at den etablerte kjønnsidentitet er irreversibel etter puberteten … Konflikten må derved søkes redusert på annet vis, vanligvis gjennom hormonell/kirurgisk kjønnsskiftebehandling.”

26 Berthold Grünfeld to Fredrik Mellbye, 25 June 1981, Oslo helseråd, box 122, Homofile – transseksualitet, OCA. “… ut fra deres subjektive opplevelse av sin situasjon.”

27 The Oslo Health Council, December 1979, “Utredning om transseksualitet” [Report on transsexuality], 5, Oslo helseråd, box 122, Homofile – transseksualitet, OCA. “Vi anser det som viktig at alle potensielle behandlere tidlig kommer i kontakt med klienten og at vurderingen skjer med synspunkter fra alle ovennevnte fagområder.”

28 Ibid., 6.

29 Ibid., 5. “Sannsynligheten for at en transseksuell vil få god tilpassing etter konvertering, øker hvis den sosiale stabiliteten før behandling er god.”

30 Ibid., 7.

31 Physician 1 interview with author, 24 October 2019. “Et krav som var veldig sterkt var at for å få kjønnsskifte, så måtte man kunne passere som det motsatte kjønn. Og det gjorde for eksempel at store menn, de fikk ikke, og de med for store sko. Jeg husker veldig godt måten dette passeringskravet ble omtalt. Snakk om kulturell utforming av maskulinitet og femininitet og hva som er riktig og galt og normalt og unormalt. Det er veldig underlig å tenke på i dag, synes jeg.”

32 Hanna interview with author, November 2019 (“Hanna” is a pseudonym). “… et veldig konservativt syn på hvordan en kvinne skulle se ut og være. Om du hadde et litt for maskulint uttrykk, så vet jeg at det var flere som ble stoppet. De som var for høye, ble stoppet.”

33 Surgeon, case 1007, medical records, Oslo University Hospital Archive (hereafter OUHA). “Hun virket ikke umiddelbart overbevisende somatisk sett, med en stor grov legemsbygning inklusive et stort grovt ansikt med rikelig mørk skjeggvekst, men det kan synes opplagt at hun definitivt har valgt kvinnerollen og kan gjennomføre denne og da er det vel riktig å hjelpe til med kirurgi.”

34 Psychologist 1 interview with author, 31 October 2019.

35 The Oslo Health Council, December 1979, “Utredning om transseksualitet” [Report on transsexuality], 2, Oslo helseråd, box 122, Homofile – transseksualitet, OCA. “… en hjelp til bedre tilpasning.”

36 The Counselling Service for Homosexuals, 1977, case 1007, medical records, OUHA. “Klienten befinner seg i en svært komplisert psykisk og sosial situasjon. Han [*sic*] har helt siden barneårene hatt behov for å kle seg, og å opptre som en kvinne. På grunn av dette har han aldri fått fotfeste i noen yrkessituasjon eller situasjon som har gitt alminnelig sosial status/akseptasjon.”

37 Ibid. “… å starte med å få kartlagt hvilke reelle muligheter han har for attføring/yrke.”

38 Contrast this with the health needs of people who request medical transition without social transition, see (Rachlin 2018).

39 Hanna interview. “En av de tingene de vektla, de var alle opptatt av en ting, det var hva slags støtte har du fra familien. Til og med kirurgen spurte hva sier mamma, pappa og søsken. … Jeg kom ikke videre til vurdering som pasient hos Grünfeld med mindre jeg hadde fortalt det. Det var et ikke-diskuterbart tema.”

40 Hanna interview. “Jeg fikk inntrykk av at han skulle ikke ha det på seg at hele min slekt tok avstand fra meg uten at jeg hadde gjort et forsøk på å gjøre noe med det. Og han mente at det hadde en utrolig stor verdi i det sosiale, så i de sosiale diskusjonene var det jeg hadde gjort med familien et tilbakevennende tema, ferieturer med søsken som var på besøk, besøke slektninger. … Han vektla det sosiale livet du hadde som kvinne ekstremt mye. Det var der han hentet spørsmål om hvordan jeg håndterte livet, daglige situasjoner og fest og moro.”

41 Psychologist 1 interview. “Jeg syntes det var ganske vanskelig å si nei. Hvem var jeg som skulle bestemme over deres liv?”

42 Ibid. “Jeg ville at de ikke skulle bli så utrerte, mer på skrullesiden. De ville gjerne hete Sonja og Sylvia. Jeg prøvde å få dem til å velge navn som var litt mer i nærheten av det de hadde.”

43 I am grateful to the reviewer for pointing this out.

44 Psychologist 1 interview. “Jeg ville at de skulle ta med seg noe av sin identitet på en måte. Det var litt sårt at de skulle tro at det skulle bli en fullstendig transformasjon. Jeg tenkte at de hadde en del bra med seg fra før, at de ble ikke helt nye.”

45 Hanna interview. “Han ville at vi skulle ha med oss hele mennesket. Vi skulle ha med oss hele historien. Alt det du hadde med deg fra før, du skulle ikke legge noe bak deg.”

46 See forthcoming paper by the author for how psychiatry took control over the field of trans medicine at the turn of the century.

47 For Norway, see (Slagstad 2020). For the United States, see for instance (Epstein 1996). For the history of transgender activism, see (Meyerowitz 2002; Stryker 2017).

48 “Den tilsløres ved hjelp av såkalt faglig resonnement.”

49 Psychologist 2 interview with author, Oslo 20 January 2020. “Jeg følte veldig at jeg/vi ivaretok pasientenes situasjon, følelser, integritet. At man ikke skulle gjøre vondt verre, at man ikke skulle sette i gang uten at det var ordentlig fundament for det.”

50 See also (Mol et al. 2010). For the contrast between “patients’ rights” and “care” in mental healthcare, see (Pols 2003).

51 See also (Campbell and Stark 2015).

52 “Våre klienter forteller om en sterk desperasjon over å leve i et samfunn der kvinner ikke har lov til å se ut som menn og omvendt. For våre klienter oppleves disse grensene så snevre at bare en operasjon kan løse konflikten mellom andres forventninger og egen adferd.”

53 “Slike tilbud medvirker i en større sammenheng til å opprettholde de snevre rammene for kjønnsrolleadferd og de strenge kravene om kjønnsrollekonformitet som vi har omtalt.”

54 There is a vast historiography on the emergence of epidemiology and risk-factor thinking in preventive medicine following the epidemiological transition, i.e., the shift from mortality from infections to chronic, non-infectious causes. See for instance (Oppenheimer 2006; Brandt 1990). For the role of pharmaceuticals in this process, see (Greene 2007).

55 The AIDS crisis represented a turning point in which the structural conditions and social determinants of health inequities again came into public health focus. For an early pivotal collection of essays, see (Fee and Fox 1988). The legacies of social justice-oriented approaches to health and illness could nonetheless be seen as part of the broader social medicine movement in the United States, even though advocates and activists often worked outside the formal academic institutions of social medicine.
